# Natural Language Processing and Social Determinants of Health in Mental Health Research: AI-Assisted Scoping Review

**DOI:** 10.2196/67192

**Published:** 2025-01-16

**Authors:** Dmitry A Scherbakov, Nina C Hubig, Leslie A Lenert, Alexander V Alekseyenko, Jihad S Obeid

**Affiliations:** 1 Biomedical Informatics Center, Medical University of South Carolina, Charleston, SC, United States; 2 Interdisciplinary Transformation University, Linz, Austria

**Keywords:** natural language processing, datasets, mental health, automated review, depression, suicide, mental health research, NLP, artificial intelligence, AI, scoping review, determinant, large language model, LLM, quantitative, automation

## Abstract

**Background:**

The use of natural language processing (NLP) in mental health research is increasing, with a wide range of applications and datasets being investigated.

**Objective:**

This review aims to summarize the use of NLP in mental health research, with a special focus on the types of text datasets and the use of social determinants of health (SDOH) in NLP projects related to mental health.

**Methods:**

The search was conducted in September 2024 using a broad search strategy in PubMed, Scopus, and CINAHL Complete. All citations were uploaded to Covidence (Veritas Health Innovation) software. The screening and extraction process took place in Covidence with the help of a custom large language model (LLM) module developed by our team. This LLM module was calibrated and tuned to automate many aspects of the review process.

**Results:**

The screening process, assisted by the custom LLM, led to the inclusion of 1768 studies in the final review. Most of the reviewed studies (n=665, 42.8%) used clinical data as their primary text dataset, followed by social media datasets (n=523, 33.7%). The United States contributed the highest number of studies (n=568, 36.6%), with depression (n=438, 28.2%) and suicide (n=240, 15.5%) being the most frequently investigated mental health issues. Traditional demographic variables, such as age (n=877, 56.5%) and gender (n=760, 49%), were commonly extracted, while SDOH factors were less frequently reported, with urban or rural status being the most used (n=19, 1.2%). Over half of the citations (n=826, 53.2%) did not provide clear information on dataset accessibility, although a sizable number of studies (n=304, 19.6%) made their datasets publicly available.

**Conclusions:**

This scoping review underscores the significant role of clinical notes and social media in NLP-based mental health research. Despite the clear relevance of SDOH to mental health, their underutilization presents a gap in current research. This review can be a starting point for researchers looking for an overview of mental health projects using text data. Shared datasets could be used to place more emphasis on SDOH in future studies.

## Introduction

Natural language processing (NLP) has emerged as a valuable tool in mental health research, offering innovative ways to extract and analyze information from various sources. Studies have shown the feasibility of using NLP in extracting evidence of online gaming and internet use from electronic health records (EHRs) in adolescent mental health patients [1]. NLP applied to clinical notes has been found to more accurately identify mental illness and substance use among people living with HIV compared to structured EHR fields alone [2]. Furthermore, NLP in healthcare enables the transformation of complex narrative information into valuable products like clinical decision support and adverse event monitoring in real-time via EHRs [3,4].

Outside of EHRs, NLP techniques have been used to make inferences about individuals’ mental states based on their social media posts [5]. Additionally, NLP, coupled with machine learning approaches, has shown promising performance in tasks such as text classification and sentiment mining in mental health contexts [6]. The application of NLP extends to identifying work-related stress among health professionals, highlighting its versatility in diverse health care settings [7].

In the context of mental health disorders like schizophrenia, schizoaffective disorder, and bipolar disorder, NLP applied to EHRs offers opportunities to create large datasets for research purposes [8]. Furthermore, NLP has been used to increase prediction accuracy and reduce subgroup differences in personnel selection decisions, showcasing its value in improving decision-making processes [9].

At the same time, getting access to text datasets for NLP analysis is challenging for many researchers. Many of the datasets have strict privacy and personal data protection policies restricting access to the data for third-party researchers. This hinders research and introduces the problem of reproducibility since the results of the studies cannot be verified by unaffiliated investigators. One of the aims of this review is to compile a collection of datasets that are available to the mental health research community, which, in turn, may facilitate research in the field of mental health.

Another potential problem with research using NLP for mental health is insufficient consideration of social determinants of health (SDOH) information during the analysis. The association between social determinants and mental health outcomes is well-established, with factors such as poverty, inequality, stigma, discrimination, and social exclusion identified as significant contributors to mental health burdens [10,11]. NLP has become a valuable tool for extracting SDOH from sources like clinical notes, social media, and EHR in health care research [12-15]. Evaluating the use of SDOH in NLP projects for mental health is another goal behind this review.

To our knowledge, no previous study has examined the range of NLP datasets and the inclusion of SDOH data in research projects that use NLP for mental health. We have opted for a scoping review following the guidelines outlined by Arksey and O’Malley [16]. The goals of this scoping review are to review and summarize the literature on (1) the variety of mental health areas that leverage NLP, (2) information on the types of text datasets used in these projects and whether they are sharable, and (3) the extent to which SDOHs are used or investigated in these projects.

A novel aspect in this scoping review is the use of large language models (LLMs), a subfield of generative artificial intelligence (AI), to automatically parse a large volume of citations to find relevant studies and extract information under the minimal supervision of a human reviewer. A recent statement by the National Institute for Health and Care Excellence highlights the potential of AI in the systematic review process automation [17].

## Methods

### Study Design

This study was created and revised following the recommendation of PRISMA-ScR (Preferred Reporting Items for Systematic Reviews and Meta-Analyses extension for Scoping Reviews) and updated JBI (formerly known as Joanna Briggs Institute) guidance for the conduct of scoping reviews [16,18-20]. The completed PRISMA-ScR checklist can be found in Multimedia Appendix 1.

### Inclusion and Exclusion Criteria

All publications were considered if they did not meet one or more of the exclusion criteria. Citations were excluded if:

- They did not use any type of NLP, such as transformers, pattern-matching (eg, regular expressions), ChatGPT, GPT-3, Bidirectional encoder representations from transformers, Llama, Mistral, other LLMs, latent Dirichlet allocation and latent semantic analysis, deep learning or machine learning applied to text, and similar.
- They were not focused on one of the mental health areas, such as psychology, well-being, psychiatry, social work, substance abuse, marriage therapy, addiction therapy, suicide, grief, bereavement, trauma, stressful life events, or counseling.
- They were review papers (systematic, scoping, literature, narrative, and other type of reviews), conference papers, or book chapters.
- They were not related to human health or well-being.
- PDF file of the full-text publication could not be located automatically using Covidence (Veritas Health Innovation), EndNote (Clarivate), and Zotero (Corporation for Digital Scholarship).

### Search Strategy

The initial search was conducted in September 2024 in PubMed, Scopus, and CINAHL Complete databases using title and abstract search filters. The search strategy (designed by DAS and JSO) was broad enough to capture different NLP and machine-learning methods related to mental health. Textbox 1 presents the search query for the databases.

Textbox 1. **Search strategy for PubMed and Scopus.**

(“natural language processing” OR “large language model*” OR “LLM” OR “NLP” OR “ChatGPT” OR “GPT-3” OR “GPT-4” OR “Llama” OR “Mistral” OR “BARD” OR “Mixtral” OR “transformer*” OR “Gemini” OR “Copilot” OR “BERT” OR “RoBERTa” OR “ALBERT” OR “Claude” OR “text mining” OR “text extraction” OR “Generative AI” OR “Natural language understanding” OR “GLoVe” OR “text2vec” OR “doc2vec” OR “word2vec” OR “fastText” OR “attention mechanism” OR “sequence-to-sequence models” OR ((“CNN” OR “neural network*” OR “GRU” OR “Gated Recurrent Unit” OR “Long Short-Term Memory” OR “RNN” OR “LSTM” OR “DNN” OR “deep learning” OR “SVM” OR “support vector machine*” OR “gradient boosting” OR “LASSO” OR “XGBoost” OR “AdaBoost” OR “random forest” OR “regression” OR “machine learning”) AND “text”)) AND (“mental” OR “well-being” OR “depression” OR “anxiety” OR “social work” OR “psychology” OR “psychiatry” OR “abuse” OR “violence” OR “addiction” OR “suicide” OR “grief” OR “bereavement” OR “trauma” OR “stressful life events” OR “counseling”) AND (“database” OR “dataset” OR “repository” OR “corpus” OR “collection” OR “reports” OR “discharge summaries” OR “documents” OR “records” OR “patient summaries” OR “notes” OR “text” OR “texts”)

### Screening and Extraction Process: LLM Assistance

**Figure 1.**
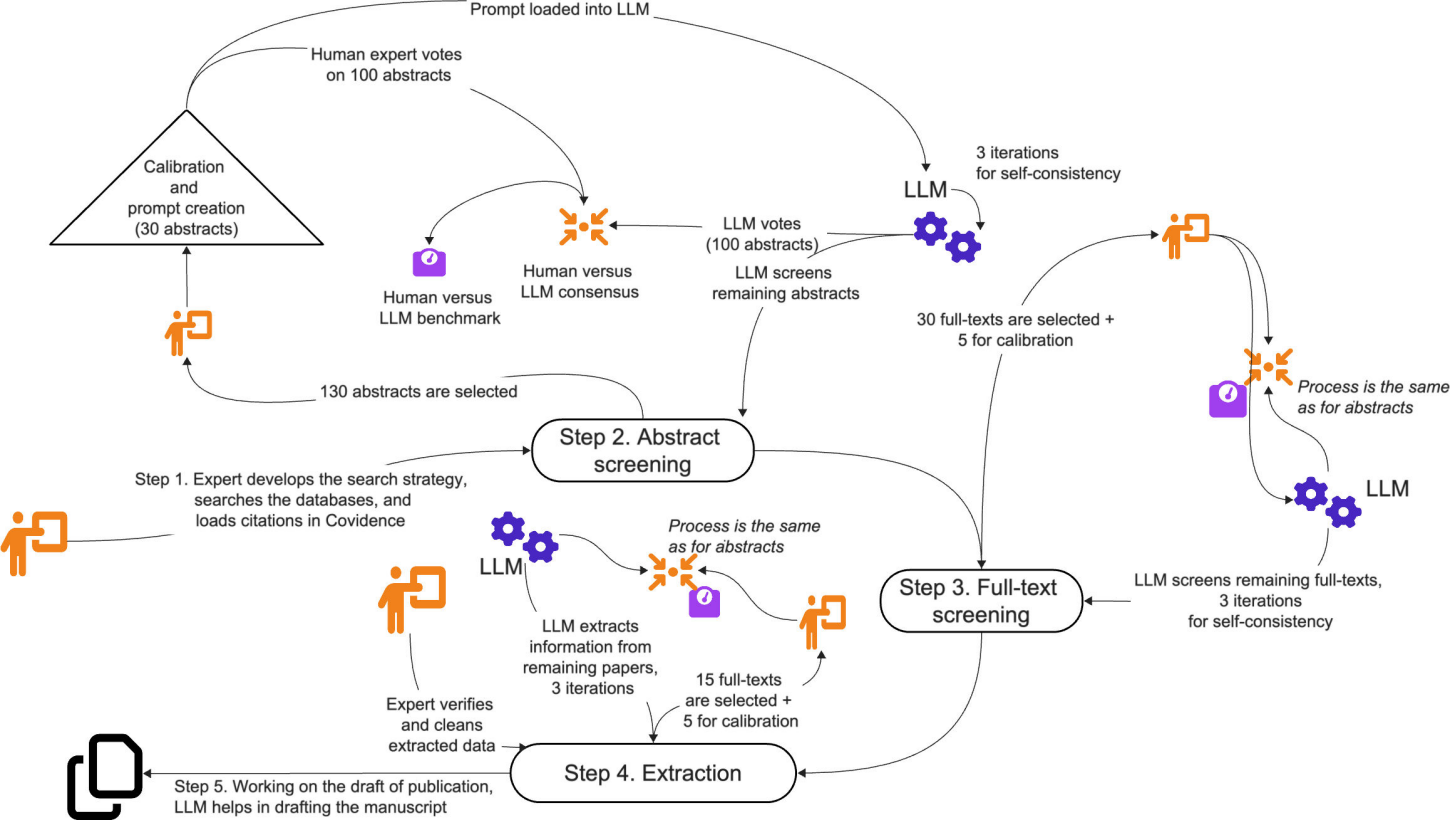
LLM add-on assistant in Covidence screening. LLM assists in the abstract screening, full-text screening, extraction, and document drafting phases. Both human and LLM screen and/or extract data using a subset of abstracts/full texts, human compares their results and reaches a consensus (“LLM consensus” in the figure), and the benchmarks of both—human and the LLM—against this consensus are recorded. LLM: large language model.

All citations were uploaded to Covidence, which was used to track the progress of the project in lieu of the protocol. The screening and extraction process took place in Covidence. The specific method we used to conduct this review was automating the process of screening and extraction with the help of the LLM module for Covidence that we developed. The process of using LLM for screening and extraction is depicted in Figure 1.

Our LLM module works by interacting with Covidence using R scripts with the Selenium automation package [21]. Our scripts, which can run both locally or on a server, pass content between Covidence and an Azure OpenAI LLM (Microsoft Corporation) using the application programming interface wrapped in the Python “openai” library [22]. The models used in this study were GPT-4o and GPT-4o-mini. Once the model generates the response, our add-on module automates actions in Covidence, such as clicking the Include/Exclude buttons. Our automation scripts require a PDF file of the full text to be uploaded into Covidence for full-text screening and extraction. The LLM module supports non-English languages natively. Our software code used in this module is still under development and has not yet been made public; however, the LLM prompts for each phase of the review are provided in Multimedia Appendix 2. A similar approach was described in our recent paper on the utility of LLMs in literature reviews [23].

The resulting process can be described as follows. Each of the three main stages of review in Covidence (abstract screening, full-text screening, and extraction) included (1) a calibration phase, where a human reviewer (DAS) experienced in conducting scoping and systematic reviews screened a small sample of abstracts or full-text studies to get a deeper understanding of inclusion criteria and extraction categories; (2) we then created a prompt for the LLM and tested the LLM performance on another sample of abstracts or full texts; and (3) three repeated requests to the LLM with the same prompt were automated using software scripts, and the majority vote principle was used to determine the LLM vote (eg, LLM votes for an abstract: “include,” “exclude,” “include” means LLM final vote is “include”). Out of these three requests, two were made using GPT-4o-mini and one using more powerful GPT-4o for screening; all three requests used GPT-4o-mini for extraction to cut application programming interface costs.

During the benchmark, a human reviewer (DAS) first compared his votes against the LLM votes to produce a new reference set of labels (called “human-LLM consensus”). This step is required because LLM can detect cases missed by a human. Then, both the initial human reviewer’s (DAS) and LLM votes were measured against the human-LLM consensus labels. Extraction precision was measured using a simplified benchmark where the LLM results on 30 publications were checked by a human reviewer (DAS) for precision only. The benchmarks are provided in Multimedia Appendix 3.

### Data Extraction Categories

The data charting form for extraction was designed by human experts (DAS and JSO) and adopted into the LLM prompt to collect the following primary information:

- Author, year, title
- Country or US state (if it is in the United States) where the study was conducted or first author’s affiliation location
- NLP method that was used (generally described in the Methods section), for example, recurrent neural network, convolutional neural network (CNN), random forest, deep learning, pattern-matching, ChatGPT, and GPT-4
- What mental health problem or problems were investigated in the paper?
- What is the mental health area or specialty that best represents this paper (psychology, well-being, psychiatry, social work, substance abuse, marriage therapy, addiction therapy, suicide, grief, bereavement, trauma, stressful life events, counseling, other)?
- Variables used in the study related to demographics, for example, age, race, ethnicity, gender, sex at birth, marital status, relationship status, and sexual orientation
- Variables used in the study related to SDOH, such as none mentioned, urban or rural, transportation availability, access to health care, incarceration, income, poverty, health insurance, language knowledge, living arrangement, children or childless, family, adverse childhood experiences, housing, education, religion, stress, traumatic events, and stressful life events
- Name of the text dataset that was used in the study
- What is the type of this text dataset (eg, clinical notes, therapy session notes, social media platforms, web forum, other)?
- What information or variables were extracted from this text dataset?
- Is it mentioned in the paper if other researchers can get access to this text dataset?
- If it is mentioned in the paper that it is possible to get access to this text dataset, what kind of access is it (eg, public, public with restrictions, private, not given, not mentioned)? If the dataset can be found on the web or in well-known competition platforms like Kaggle, it is considered public
- If it is mentioned in the paper that access to this text dataset is public or public with restrictions, what is required to get access (can be training, signing a use agreement, emailing the author, or similar)?
- A URL to the text dataset, if provided. The returned URL was validated using an R script to test if an “OK” reply is returned by the server.

Extracted results were synthesized using a table with a complete list of all citations, using maps for location information and column plots displaying frequency statistics for other extracted variables.

### Data Cleaning and Paper Drafting

ChatGPT with the GPT-4o model was used to clean the extraction data, specifically, format the case, remove duplicates, and sort entries into higher-level groups. Scite.ai (Research Solutions) was used to draft parts of the *Introduction* and *Discussion* sections, while ChatGPT was used to draft the abstract and *Results* section of this paper by generating text and R code snippets. All output generated by the LLMs was verified, reviewed, and edited by the authors. Due to the significant number of reviewed citations, publication information, such as authors, title, and DOI, is provided in Multimedia Appendix 4.

## Results

**Figure 2.**
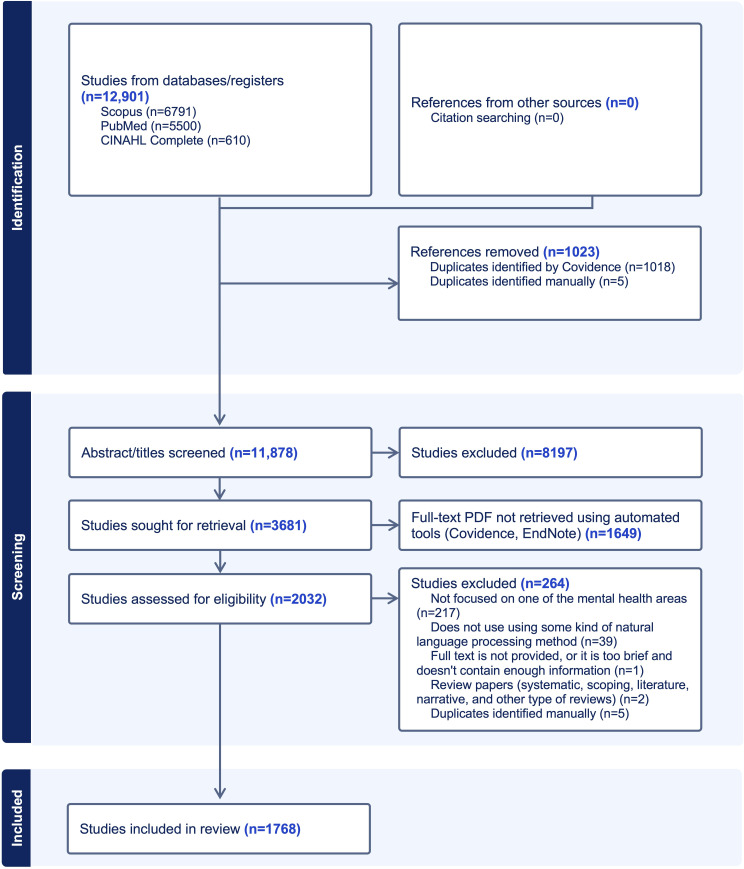
Shows the flow diagram of the scoping review process. Initially, 12,901 studies were identified from various databases and registers: 6791 from Scopus, 5500 from PubMed, and 610 from CINAHL Complete. After the removal of 1023 duplicates by Covidence, 11,878 studies were retained for screening.

During the abstract and title screening phase, 8197 studies were excluded based on the exclusion criteria, leaving 3681 studies for retrieval. Out of these, 1649 studies could not have their full-text PDFs retrieved using automated tools such as Covidence or EndNote. Consequently, 2032 studies were assessed for eligibility. Of these, 264 studies were excluded for the following reasons: 217 studies were not focused on one of the mental health areas, 39 studies did not use any NLP methods, 1 study was too brief and lacked sufficient information, and 2 studies were review papers (systematic, scoping, literature, narrative, or other types of reviews), and 5 additional duplicates were identified during full-text screening. The final review included 1768 studies. The flow diagram of the scoping review process is shown in Figure 2.

**Figure 3.**
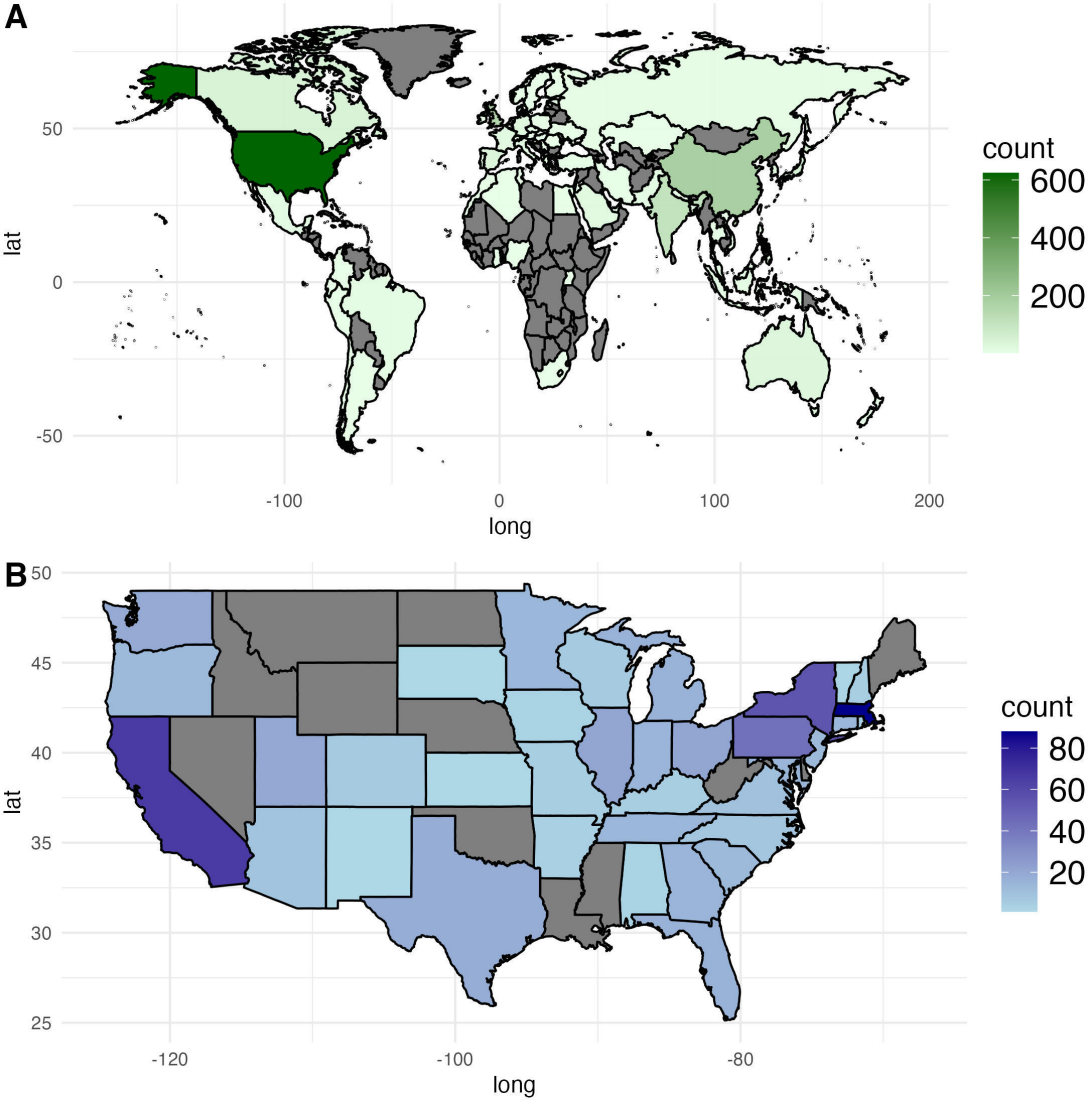
(A) Number of publications by country of origin. (B) Number of publications by state in the United States. Darker shades represent locations with more publications. Grey color means the absence of publications from a given location.

Figure 3 illustrates the geographic distribution of 1768 studies reviewed in this analysis. Most studies originated from the United States, with 624 (35.3%) studies. China contributed 197 (11.1%) studies, followed by the United Kingdom (n=167, 9.4%) and India (n=120, 6.8%).

Canada contributed 51 (2.9%) studies. Other notable contributors include Japan with 49 (2.8%) studies, Spain with 39 (2.2%) studies, Australia with 38 (2.1%) studies, South Korea (n=27, 1.5%), Germany (n=26, 1.5%), the Netherlands (n=25, 1.4%), Saudi Arabia (n=24, 1.4%), Italy (n=22, 1.2%), and France (n=21, 1.2%), and several other countries each contributing between 1 and 16 studies.

Within the United States, Massachusetts led the contributions with 88 (14.1%) studies, followed by California with 66 (10.6%) studies. New York contributed 55 (8.8%) studies, while Pennsylvania provided 43 (6.9%) studies. Ohio and Illinois each contributed 21 (3.4%) studies. Other states, such as Utah, Washington, and Texas, contributed 20 (3.2%), 19 (3%), and 18 (2.9%) studies, respectively. Michigan and Florida each added 16 (2.6%) studies, while Maryland, Georgia, and Indiana each contributed 15 (2.4%) studies. Tennessee, Minnesota, and Connecticut each contributed 14 (2.2%) studies, followed by New Jersey, Oregon, and South Carolina with 13 (2.1%) studies each. Other states contributed fewer than 10 studies.

**Figure 4.**
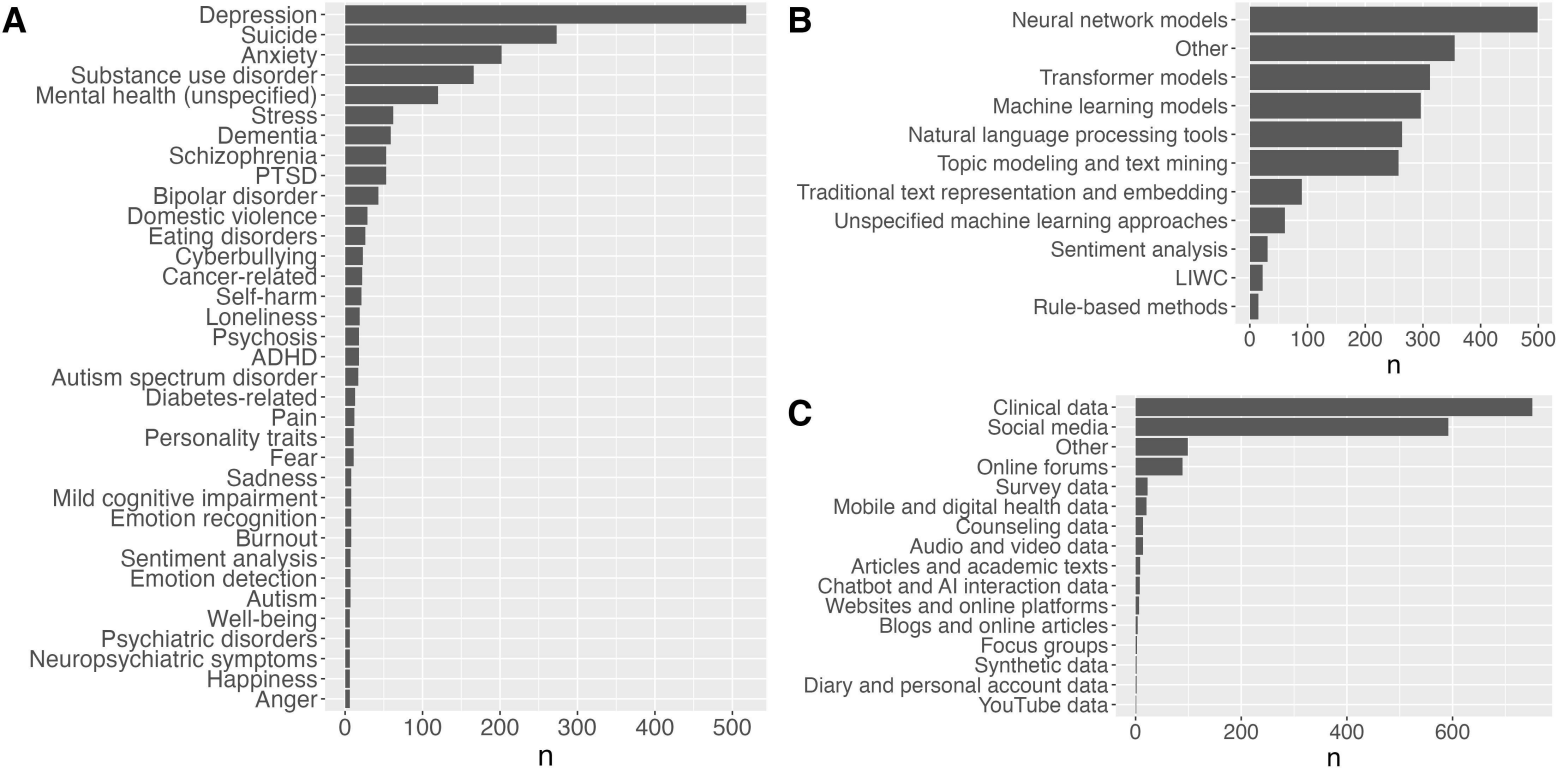
(A) Mental health outcomes studied in reviewed publications (mentioned in ≥5 citations). (B) NLP methods or tools used (mentioned in ≥5 citations). (C) Types of datasets used for analysis. The x-axis represents the number of publications featuring a given outcome (A), method (B), or dataset type (C). ADHD: attention-deficit/hyperactivity disorder; AI: artificial intelligence; LIWC: linguistic inquiry and word count; NLP: natural language processing; PTSD: posttraumatic stress disorder.

Figure 4A illustrates the various mental health topics covered in the reviewed papers. The most frequently discussed topic is depression, which is covered in 518 (29.3%) papers. This is followed by suicide, featured in 273 (15.4%) papers, and anxiety, discussed in 202 (11.4%) papers. Substance use disorder is also a significant topic, appearing in 166 (9.4%) papers, while mental health (unspecified) is mentioned in 120 (6.8%) papers.

Other notable topics include stress (n=62, 3.5%), dementia (n=59, 3.3%), posttraumatic stress disorder (n=53, 3%), and schizophrenia (n=53, 3%). Bipolar disorder appears in 43 (2.4%) papers, and domestic violence is discussed in 29 (1.6%) papers. Eating disorders are mentioned in 26 (1.5%) papers, while cyberbullying and cancer-related topics are covered in 23 (1.3%) and 22 (1.2%) papers, respectively. Self-harm is discussed in 21 (1.2%) papers, and loneliness is covered in 19 (1.1%) papers.

Additionally, other mental health issues such as attention-deficit/hyperactivity disorder (n=18, 1%), psychosis (n=18, 1%), autism spectrum disorder (n=17, 1%), and diabetes-related mental health issues (n=13, 0.7%) are also represented. Topics such as pain (n=12, 0.7%) and fear (n=11, 0.6%) are covered, along with personality traits (n=11, 0.6%) and burnout (n=8, 0.5%). A variety of other mental health topics appear in 1‐7 papers.

Figure 4B illustrates the various NLP methodologies and tools discussed in the papers. The most frequently mentioned are neural network models (n=499, 28.2%), which include examples such as CNN, long short-term memory (LSTM), Bidirectional LSTM-CNN (BI-LSTM-CNN), gated recurrent unit, and recurrent neural network. Other machine learning models are discussed in 289 (16.3%) papers, highlighting the use of random forest, support vector machine, regression models, and gradient boosting trees.

Transformer models appear in 312 (17.6%) papers, examples include Bidirectional encoder representations from transformers, GPT-3, LLAMA-2, and Roberta. NLP tools are featured in 264 (14.9%) papers, which use tools such as Spacy NLP Library, Stanford CoreNLP, and GATE for processing and analyzing text data. Topic modeling and text mining are discussed in 258 (14.6%) papers, using techniques such as latent Dirichlet allocation, structural topic modeling, and biterm topic modeling for extracting themes and patterns from text data.

Traditional text representation and embedding methods are mentioned in 90 (5.1%) papers, including methods such as term frequency-inverse document frequency, Word2Vec, and N-gram representation. Unspecified machine learning approaches appear in 61 (3.4%) papers, while sentiment analysis is discussed in 31 (1.8%) papers. Finally, linguistic inquiry and word count (LIWC) is mentioned in 22 (1.2%) papers, showcasing tools such as LIWC15 Text Analysis and LIWC Dictionaries. Rule-based methods are included in 15 (0.8%) papers.

Sentiment analysis is discussed in 30 (1.9%) papers, with approaches such as Valence Aware Dictionary and sEntiment Reasoner, aspect-based sentiment analysis, and text sentiment analysis. Rule-based methods are featured in 15 (1%) papers, using approaches such as pattern-matching and lexicon-based NLP to perform specific text-processing tasks based on predefined rules. Finally, Bayesian models are mentioned in 3 (0.2%) papers, where techniques, for example, Bayesian networks and Bayesian logistic regression, are applied, indicating a more niche focus on this approach within the reviewed literature. The other category, covered in 355 (20.1%) papers, represents a wide range of techniques beyond the most common methods, including various specialized or less frequently used approaches.

Figure 4C presents an overview of the types of datasets used in the reviewed studies. The most commonly used dataset type is clinical data, which appears in 751 (42.4%) papers, followed by social media datasets with 592 (33.4%) papers. Web forums have some representation as well, with 89 (5%) papers, and the other category comprises 99 (5.6%) papers. Survey data is also notable, appearing in 23 (1.3%) papers, while mobile and digital health data is used in 21 (1.2%) papers.

Less frequently used datasets include counseling data (n=14, 0.8%), audio and video data (n=14, 0.8%), and chatbot and AI interaction data (n=8, 0.5%). The studies and academic texts category is represented in 9 (0.5%) papers, while websites and web platforms account for 7 (0.4%) papers. Blogs and web studies have 4 (0.2%) papers.

Other datasets, such as diary and personal account data and synthetic data, each appear in 2 (0.1%) papers, along with focus groups, which are represented in 3 (0.2%) papers. Finally, YouTube data is noted in 1 (<0.1%) paper, indicating niche areas of study or emerging methodologies within the broader field.

**Figure 5.**
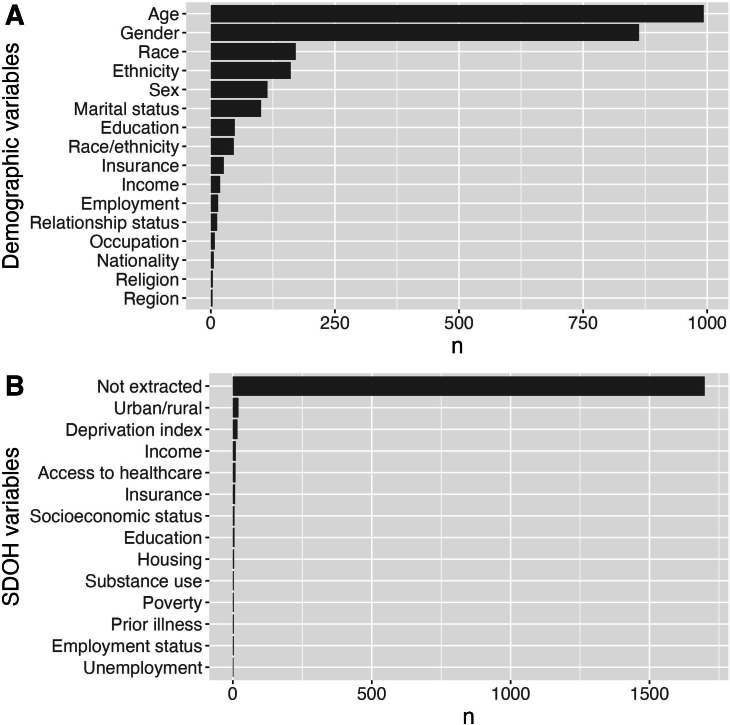
(A) Demographic variables used in the studies (with ≥3 mentions). (B) Social determinants used in the studies (≥2 mentions). The x-axis represents the number of publications featuring a given social determinant (A) or demographic variable (B). SDOH: social determinants of health.

Figure 5A presents a detailed overview of the demographic variables frequently used in the reviewed studies. Age is the most commonly extracted variable, appearing in 993 (56.2%) studies. Following closely is gender, featured in 863 (48.8%) studies. Other significant demographic variables include race (n=171, 9.7%) and ethnicity (n=161, 9.1%). Sex is documented in 114 (6.4%) studies, while marital status appears in 101 (5.7%) studies.

Less frequently reported variables include education (n=48, 2.7%), race or ethnicity (n=46, 2.6%), and insurance (n=26, 1.5%). Income is mentioned in 19 (1.1%) studies, with employment in 15 (0.8%) studies, relationship status in 13 (0.7%) studies, and occupation in 8 (0.5%) studies. More specialized demographic insights are provided by variables, for example, nationality (n=6, 0.3%), religion (n=4, 0.2%), and region (n=3, 0.2%). Additionally, niche variables such as aboriginal status, career, and socioeconomic status are noted, each appearing in 2 (0.1%) studies.

Figure 5B offers an overview of the SDOH variables used in the reviewed studies. The urban or rural status is the most frequently reported variable, appearing in 20 (1.2%) studies. Following closely is the deprivation index, included in 17 (1.1%) studies. Income is mentioned in 11 (0.7%) studies, underscoring its significance in assessing economic conditions.

It is important to highlight that demographic variables had a notable number of false positives during extraction, with a precision rate of 0.66, suggesting that the actual counts for gender and age may be significantly lower.

The relevance of access to health care and insurance is reflected in their occurrence in 9 (0.5%) and 8 (0.5%) studies, respectively. Education and socioeconomic status are recorded in 6 (0.4%) studies each, while housing is featured in 5 (0.3%) studies.

Less frequently reported variables include poverty and substance use, each appearing in 4 (0.3%) studies, as well as employment status and prior illness, each in 3 (0.2%) studies. Additional variables such as unemployment (n=2, 0.1%) and various specific factors—for example, domestic violence, drug involvement, and others—are noted in just 1 (0.06%) study each.

Table 1 shows the most frequently extracted information from the text datasets. The scope of extracted data includes information related to sentiments and emotions, health conditions, health symptoms, personality traits, violence and bullying, suicide indicators, user engagement (likes, shares), survey data, and language features, to name a few.

A significant majority of studies (n=911, 51.5%), did not clarify whether their datasets were accessible, while 857 (48.5%) studies included access information for their datasets. Regarding the specific levels of access, the vast majority of studies fell into the “unclear” category, with 1128 (63.7%) studies failing to provide explicit information about dataset accessibility. In contrast, 362 (20.5%) studies indicated that their datasets were publicly accessible, while 263 (14.9%) studies allowed public access with certain restrictions, thus enabling data use under specific conditions. Only a minimal number of studies categorized their datasets as private, with just 9 (0.5%) studies restricting access to particular individuals or groups. Additionally, 4 (0.2%) studies did not provide any information regarding their dataset access levels.

Additional information on the text datasets, access levels, links, and other extracted information can be found in Multimedia Appendix 4.

**Table 1.** Information extracted from text datasets (terms with more than 10 mentions are displayed).

Extracted term	Frequency, n
Neutral	34
Sadness	33
Negative	30
Sentiment scores	28
Fear	25
Symptoms	24
Linguistic features	22
Sentiment	21
Demographic information	20
Demographics	20
Disgust	20
Anger	18
Tweets	18
Surprise	17
Emotions	16
Gender	16
Anxiety	15
PHQ-8 scores	15
Sentiments	14
Age	13
Comments	13
Depression	12
Joy	12
Keywords	12
Medications	12
Suicidal ideation	12
Valence	12
Arousal	11
Diagnoses	11
Demographic characteristics	10
EEG signals	10
Emotional states	10
Guilt	10
Topics	10

## Discussion

### Principal Findings

Our LLM-assisted scoping review revealed a wide range of projects related to mental health topics that use NLP. The United States was the dominant source of publications, with more than a third of all publications, but China, the United Kingdom, and India follow closely behind, reflecting the worldwide interest in the application of NLP to mental health problems. The United States has a significant concentration of funding and resources dedicated to mental health research, which is not as prevalent in low- and middle-income countries with financial constraints and inequities in health care resources [24,25]. The disproportionate share of high-income countries in our review is noted by other authors as “the 90:10 research gap,” where 90% of mental health research focuses on the 10% of the global population residing in high-income countries, including the United States [26].

Depression emerged as the top mental health problem investigated, reflecting the current trends: a study by Wang et al [27] using Google Trends data in the United States reported a 61% increase in depression prevalence from 2008 to 2018. Another study by Jonson et al [28] in Sweden observed a decline in depression prevalence among 85-year-olds, potentially influenced by rising trends in younger age groups. The topic of suicide was especially well represented in our sample, highlighting the fact that suicide mortality remains a significant global public health concern. This finding is echoed by studies indicating that suicide continues to be a notable contributor to mortality worldwide [29].

Artificial neural networks appear to dominate the landscape of tools used in NLP research, with transformer models catching up in the race. Artificial neural networks represent a large and versatile category of machine learning algorithms that typically require a significant amount of training data, whether supervised or unsupervised [30]. The self-organizing, self-learning, and parallel distributed information processing capabilities of neural networks have made them invaluable in pattern recognition, signal processing, and optimization problems [31]. Moreover, artificial neural networks are recognized for their versatility in solving nonlinear problems with multiple independent variables [32], including NLP tasks.

Clinical data and social media dominate the types of datasets that were used in the reviewed papers, showing two major avenues of NLP mental health research, one with medical records data and the other with using public social media platforms.

As for SDOH and demographic variables, there is considerable overlap between the two in the extracted data. Previous work suggests that demographic variables should be part of SDOH; for example, the commonly used variable marital status reflects the social connections, stage of life, and other important social implications for individuals’ health [33]. The same can be said about age, the most frequently reported demographic variable. Research has shown that disparities in mental health outcomes persist across different age groups and are often linked to social stress, discrimination, and stigma [34]. These disparities can be exacerbated by obstacles to health care access based on factors such as ethnicity, sex, and occupation [35].

A narrative review by Shokouh et al [36] explores the idea that demographic variables could be considered an essential part of SDOH. A similar solution is to think of them as combined “sociodemographic factors” [37], where both demographic and social factors play an equally important role.

This review suggests that social and demographic factors, besides gender and age, were rarely used in the studies, highlighting a significant gap that should be addressed in future work. In addition, a manual review of LLM outputs (see Multimedia Appendix 3 for benchmark) revealed that the demographic variables category had a high rate of false positivity, which suggests that gender and age were actually used even less frequently than our numbers indicate. Most commonly, they were reported in the introduction sections as important factors and ignored in the actual analysis.

The paucity of mental health NLP studies that consider SDOH is concerning, especially considering that these factors, including stress, marital status, race, gender-based discrimination, and many others, have been shown to impact mental health outcomes [38]. One of the challenges for mental health researchers is a lack of versatile NLP tools that would allow the extraction of attributes related to SDOH and demographics. For example, while NLP models for extraction of marital status and gender exist [39,40], few models can extract a big range of SDOH at once [41], making a subgroup analysis limited to select demographic attributes (eg, age and gender). Another solution could be to extract this information from structured data. However, this is often not feasible; for example, social media metadata rarely contain information about age, region, and other sociodemographic attributes, while EHRs that contain clinical notes do not routinely collect social information [41].

Our review method proved to be sensitive at detecting relevant citations and fetched our previous work on suicide, self-harm, opioid addiction, and other topics [42-50]. LLM performance in this review surpassed the performance of a single human reviewer during the screening phase, as evident from the screening benchmarks. Reviews conducted by more than one individual human could reduce selection bias but would require significant additional research effort. Based on the estimation methodology provided by Haddaway and Westgate [51], we estimate this effort to be approximately 3500 person-hours (ie, close to a year of work for two reviewers) to conduct a review of such scope. In addition, the LLM method allowed for the inclusion of non-English papers, as LLMs are multilingual, potentially reducing the language bias; however, the performance of OpenAI GPT can vary depending on the language and the task [52], and we did not benchmark the LLM performance in other languages.

Future work could be facilitated by this review, which revealed a considerable number of shared research datasets, including URLs for some. In fact, over 600 publications disclosed the datasets they used and the level of access as public or semipublic. Disclosure of dataset use is important in research because it increases reproducibility and facilitates collaborative secondary research using existing data. A recent publication has reported that over half of the studies in psychology could not be replicated [53], which is why it is crucial to be transparent about the datasets used and the NLP methods used. Moreover, making both the code and data available to the scientific community whenever possible or providing information on why access to data is restricted is also valuable. A dataset prepared for a specific study can be used in secondary research different from the original work and may help prevent redundant data collection. A 2024 crowdsourcing challenge on HeroX was specifically aimed to “demonstrate the power of data reuse in advancing human health by proposing an impactful secondary data analysis research project” and promoted the reuse of data that has already been published.

Finding data for research is always challenging. We hope that this review can serve as a stepping stone in mental health research that leverages NLP.

### Limitations

We used LLMs, with prompts engineered using a small subset of studies, as assistants in this review project. Some extraction categories, such as demographic variables, had relatively lower accuracy, so the results of this extraction category should be taken with caution. Nevertheless, in this review, LLMs achieved remarkable results in other categories, making it possible to delegate time-consuming aspects of a literature review to the LLM, allowing researchers to spend more time on the supervision, benchmarking, and synthesis of the findings.

This study used a single human reviewer assisted by LLMs. Studies generally recommend a single-reviewer approach in some cases, such as rapid reviews [54]; however, we believe that the LLM approach could automate many of the mundane aspects of literature reviews, allowing human authors to redirect their effort toward the supervision and synthesis of the results.

Our method relies on the availability of PDFs for publications. A considerable number of papers did not have accessible PDF versions using the citation manager tools, for example, EndNote and Zotero. Thus, we had to exclude these papers from our analysis. However, we believe that the number of full-text citations that we obtained was large enough to get a statistical representation of the extracted categories and to support our observed findings discussed above.

### Conclusions

This review highlights the range of projects using NLP for mental health areas, with depression and suicide being the most frequent topics under study. Social determinants were only used in a handful of papers, with traditional demographic variables, such as age and gender, being more frequent. The extracted information could be leveraged by other researchers pursuing text datasets for mental health research projects in specific areas.

## Supplementary material

10.2196/67192 Multimedia Appendix 1 PRISMA-ScR (Preferred Reporting Items for Systematic Reviews and Meta-Analyses extension for Scoping Reviews) checklist.

10.2196/67192 Multimedia Appendix 2 Large language model prompts used for screening and extraction.

10.2196/67192 Multimedia Appendix 3 Large language model benchmarks.

10.2196/67192 Multimedia Appendix 4 Extraction table.

## Abbreviations

AI: artificial intelligence
CNN: convolutional neural network
EHR: electronic health record
LIWC: linguistic inquiry and word count
LLM: large language model
LSTM: long short-term memory
NLP: natural language processing
PRISMA-ScR: Preferred Reporting Items for Systematic Reviews and Meta-Analyses extension for Scoping Reviews
SDOH: social determinants of health
