# Supplementary material for: Natural Language Processing and Social Determinants of Health in Mental Health Research: AI-Assisted Scoping Review
Source: JMIR Ment Health. 2025 Jan 16;12:e67192. doi: 10.2196/67192 (PMC11756842; doi:10.2196/67192)
Supplement: Multimedia Appendix 1 [file mental-v12-e67192-s001.docx]

*Supplementary Appendix S1.* Preferred Reporting Items for Systematic Reviews and Meta-Analyses extension for Scoping Reviews (PRISMA-ScR) Checklist

| **SECTION** | **ITEM** | **PRISMA-ScR CHECKLIST ITEM** | **REPORTED ON PAGE #** |
| --- | --- | --- | --- |
| **TITLE** | | | |
| Title | 1 | Identify the report as a scoping review. | 1 |
| **ABSTRACT** | | | |
| Structured summary | 2 | Provide a structured summary that includes (as applicable): background, objectives, eligibility criteria, sources of evidence, charting methods, results, and conclusions that relate to the review questions and objectives. | 1-2 |
| **INTRODUCTION** | | | |
| Rationale | 3 | Describe the rationale for the review in the context of what is already known. Explain why the review questions/objectives lend themselves to a scoping review approach. | 2-4 |
| Objectives | 4 | Provide an explicit statement of the questions and objectives being addressed with reference to their key elements (e.g., population or participants, concepts, and context) or other relevant key elements used to conceptualize the review questions and/or objectives. | 3-4 |
| **METHODS** | | | |
| Protocol and registration | 5 | Indicate whether a review protocol exists; state if and where it can be accessed (e.g., a Web address); and if available, provide registration information, including the registration number. | 5 |
| Eligibility criteria | 6 | Specify characteristics of the sources of evidence used as eligibility criteria (e.g., years considered, language, and publication status), and provide a rationale. | 4 |
| Information sources* | 7 | Describe all information sources in the search (e.g., databases with dates of coverage and contact with authors to identify additional sources), as well as the date the most recent search was executed. | 4 |
| Search | 8 | Present the full electronic search strategy for at least 1 database, including any limits used, such that it could be repeated. | 5 |
| Selection of sources of evidence† | 9 | State the process for selecting sources of evidence (i.e., screening and eligibility) included in the scoping review. | 6-7 |
| Data charting process‡ | 10 | Describe the methods of charting data from the included sources of evidence (e.g., calibrated forms or forms that have been tested by the team before their use, and whether data charting was done independently or in duplicate) and any processes for obtaining and confirming data from investigators. | 7 |
| Data items | 11 | List and define all variables for which data were sought and any assumptions and simplifications made. | 7-8 |
| Critical appraisal of individual sources of evidence§ | 12 | If done, provide a rationale for conducting a critical appraisal of included sources of evidence; describe the methods used and how this information was used in any data synthesis (if appropriate). | Not performed |
| Synthesis of results | 13 | Describe the methods of handling and summarizing the data that were charted. | 8 |
| **RESULTS** | | | |
| Selection of sources of evidence | 14 | Give numbers of sources of evidence screened, assessed for eligibility, and included in the review, with reasons for exclusions at each stage, ideally using a flow diagram. | 8-10 |
| Characteristics of sources of evidence | 15 | For each source of evidence, present characteristics for which data were charted and provide the citations. | 9-11, Supplemental Appendix S4 |
| Critical appraisal within sources of evidence | 16 | If done, present data on critical appraisal of included sources of evidence (see item 12). | Not performed |
| Results of individual sources of evidence | 17 | For each included source of evidence, present the relevant data that were charted that relate to the review questions and objectives. | Supplemental Appendix S4 |
| Synthesis of results | 18 | Summarize and/or present the charting results as they relate to the review questions and objectives. | 11-18 |
| **DISCUSSION** | | | |
| Summary of evidence | 19 | Summarize the main results (including an overview of concepts, themes, and types of evidence available), link to the review questions and objectives, and consider the relevance to key groups. | 18-20 |
| Limitations | 20 | Discuss the limitations of the scoping review process. | 20 |
| Conclusions | 21 | Provide a general interpretation of the results with respect to the review questions and objectives, as well as potential implications and/or next steps. | 20 |
| **FUNDING** | | | |
| Funding | 22 | Describe sources of funding for the included sources of evidence, as well as sources of funding for the scoping review. Describe the role of the funders of the scoping review. | 21 |

*From:* Tricco AC, Lillie E, Zarin W, O'Brien KK, Colquhoun H, Levac D, et al. PRISMA Extension for Scoping Reviews (PRISMAScR): Checklist and Explanation. Ann Intern Med. 2018;169:467–473. [doi: 10.7326/M18-0850](http://annals.org/aim/fullarticle/2700389/prisma-extension-scoping-reviews-prisma-scr-checklist-explanation).

JBI = Joanna Briggs Institute; PRISMA-ScR = Preferred Reporting Items for Systematic reviews and Meta-Analyses extension for Scoping Reviews.

* Where *sources of evidence* (see second footnote) are compiled from, such as bibliographic databases, social media platforms, and Web sites.

† A more inclusive/heterogeneous term used to account for the different types of evidence or data sources (e.g., quantitative and/or qualitative research, expert opinion, and policy documents) that may be eligible in a scoping review as opposed to only studies. This is not to be confused with *information sources* (see first footnote).

‡ The frameworks by Arksey and O’Malley (6) and Levac and colleagues (7) and the JBI guidance (4, 5) refer to the process of data extraction in a scoping review as data charting*.*

§ The process of systematically examining research evidence to assess its validity, results, and relevance before using it to inform a decision. This term is used for items 12 and 19 instead of "risk of bias" (which is more applicable to systematic reviews of interventions) to include and acknowledge the various sources of evidence that may be used in a scoping review (e.g., quantitative and/or qualitative research, expert opinion, and policy document).

*Supplementary Appendix S2.* LLM Prompts used for screening and extraction.

| *Phase of the review* | *LLM prompt* |
| --- | --- |
| Abstract screening | Summarize the text abstract of a full research paper (article), and given the below criteria list, say if the full paper is likely to be included, excluded, or unclear.  Criteria list.  Include: Paper should be using some kind of natural processing method (NLP), like transformers, pattern-matching, ChatGPT, GPT-3, BERT, Llama, Mistral, large language models, LDA/LSA, deep learning or machine learning applied to text, and similar.  Include: Paper should be in one of the mental health areas, such as: psychology, well-being, psychiatry, social work, substance abuse, marriage therapy, addiction therapy, suicide, grief, bereavement, trauma, stressful life events, counseling, or related. Cyberbullying and study of emotions should be included, however, aggressive and violent language should be excluded.  Exclude: If any of the Include criteria doesn't match  Exclude: Review papers (systematic, scoping, literature, narrative, and other type of reviews, but retrospective data reviews and chart reviews should be included), book chapters.  Exclude: Abstract is not provided, or it is too brief and doesn't contain enough information  Follow this format:  1) First provide some explanations why each study should be included or excluded.  2) Then format your output as follows, strictly follow this format, use equal(=) sign, if study is excluded, write 'answer=excluded', if study is included output 'answer=included', or if it is unclear write 'answer=unclear'. |
| Full-text screening | Look at the research paper (article), and given the below criteria list, say if the full paper is to be included, excluded, or unclear.  \nCriteria list.  Exclude reason 1: Doesn't use using some kind of natural language processing method (NLP), like transformers, pattern-matching, ChatGPT, GPT-3, BERT, Llama, Mistral, large language models (LLM), LDA/LSA, deep learning or machine learning applied to text, and similar.  Exclude reason 2: Not focused on one of the mental health areas, such as: psychology, well-being, psychiatry, social work, substance abuse, marriage therapy, addiction therapy, suicide, grief, bereavement, trauma, stressful life events, counseling,  Exclude reason 3: Review papers (systematic, scoping, literature, narrative, and other type of reviews, but retrospective data reviews and chart reviews should be included), conference papers, book chapters  Exclude reason 4: Not related to human health or well-being  Exclude reason 5: Full text is not provided, or it is too brief and doesn't contain enough information  \nFollow this format:  1) First provide some explanations why each study should be included or excluded.  2) Provide citation from text showing what NLP method was used and mental health problem explored.  3) Output the following, choose one best matching exclusion reason:  include=yes/no/unclear  exclude_reason=reason_number |
| Extraction of data | Definition of text dataset:  Text dataset (also called corpus, notes collection, notes database, text archive, text compilation, text repository, or similar term can be used) is a collection of texts or notes that were used for natural language processing (NLP) analysis or for training NLP models,  or for data extraction (also called text mining).  Look at the research paper (article), and extract the following information.  Follow the format given.\n  Field 1) Extract country and US state (if it is in US) where study location was. Typically it is the location where dataset comes from as described in the methods section. If this can not be determined, look at the country and US state of first author's affiliation. Output as: Country name, or USA/State Name  Field 2) What natural language processing (NLP) method was used (generally described in Methods section), example answers: the study didn't use natural language processing, word2vec, text2vec, doc2vec, RNN, CNN, SVM, random forest, deep learning, pattern-matching, ChatGPT, GPT-4, BERT, Llama, Mistral, LDA/LSA, other (provide name).  Field 3) What mental health problem(s) were investigated in the paper?  Field 4) What is the mental health area or specialty that best represents this paper, select one of: not related to mental health, psychology, well-being, psychiatry, social work, substance abuse, marriage therapy, addiction therapy, suicide, grief, bereavement, trauma, stressful life events, counseling, other (provide name).  Field 5) List all variables used in the study related to demographics, for example: age, race, ethnicity, gender, sex at birth, marital status, relationship status, sexual orientation, etc.  Field 6) List all variables used in the study related to social determinants of health, such as: none mentioned, urban/rural, transportation availability, access to healthcare, incarceration, income, poverty, health insurance, language knowledge, living arrangement, children/childless, family, adverse childhood experiences, housing, education, religion, stress, traumatic events, stressful life events, etc.  The next fields are all related to the text dataset that was used in the study:  Field 7) What is the name of the text dataset that was used for the Methods section (not to be confused with Introduction)  Field 8) What is the type of this text dataset, select one of: clinical notes, therapy session notes, social media platforms, online forum, other [insert type here]?  Field 9) What information or variables were extracted from this text dataset?  Field 10) Is it mentioned in the paper if it is possible for other researchers to get access to this text dataset?  Field 11) If it is mentioned in the paper that it is possible to get access to this text dataset, what kind of access it is? Select one of: public, public with restrictions, private, not given, not mentioned  Terms of access to text dataset can sometimes be found in the methods section, sometimes in data availability section, however this section has to specifically mention the text dataset that was used in this study. Sometimes terms of access are found in other parts of the document.  If the dataset can be found online or in well-known competition platforms like Kaggle consider access as public.  Field 12) If it is mentioned in the paper that access to this text dataset is public or public with restrictions, what is required to get access (can be training, signing use agreement, emailing the author, or similar)?  Field 13) Link (URL) to the text dataset, if provided.  Format your output as an R data.frame:  data.frame(fld1='',fld2='',fld3='',...,fld13='') |

*Supplementary Appendix S3.* LLM benchmarks

*Table 1.* Benchmark of abstract screening phase (N=100 abstracts).

|  | Sensitivity | Specificity | Pos Pred Value | Neg Pred Value | Precision | Recall | F1 | Prevalence | Detection Rate | Detection Prevalence | Balanced Accuracy |
| --- | --- | --- | --- | --- | --- | --- | --- | --- | --- | --- | --- |
| Human reviewer vs Consensus | 0.89 | 0.91 | 0.92 | 0.87 | 0.92 | 0.89 | 0.91 | 0.54 | 0.48 | 0.52 | 0.9 |
| LLM vs Consensus | 0.98 | 0.96 | 0.96 | 0.98 | 0.96 | 0.98 | 0.97 | 0.54 | 0.53 | 0.55 | 0.97 |

*Table 2.* Benchmark of full-text screening phase (N=30 full-text PDFs).

|  | Sensitivity | Specificity | Pos Pred Value | Neg Pred Value | Precision | Recall | F1 | Prevalence | Detection Rate | Detection Prevalence | Balanced Accuracy |
| --- | --- | --- | --- | --- | --- | --- | --- | --- | --- | --- | --- |
| Human reviewer vs Consensus | 0.7 | 1 | 1 | 0.87 | 1 | 0.7 | 0.82 | 0.33 | 0.23 | 0.23 | 0.85 |
| LLM vs Consensus | 0.7 | 0.95 | 0.87 | 0.86 | 0.88 | 0.7 | 0.78 | 0.33 | 0.23 | 0.27 | 0.82 |

*Table 3.* Benchmark of full-text extraction phase (N=30 full-text PDFs).

|  | Country | NLP method | Mental health Outcome | Demographic variables | SDOH  Variables | Dataset name | Dataset type | Type of information extracted | Is access to dataset discussed? | Access level mentioned | Requirements to access dataset | URL to dataset | Average |
| --- | --- | --- | --- | --- | --- | --- | --- | --- | --- | --- | --- | --- | --- |
| Precision | 0.97 | 0.9 | 0.93 | 0.77 | 0.97 | 0.9 | 0.9 | 0.87 | 0.8 | 0.8 | 0.97 | 0.87 | *0.89* |

*Supplementary Appendix S4.* The complete extraction table is available as an Excel file.

Asterisks near terms denote LLM certainty (*** match across 3 runs, ** match across 2 runs , * term matched only in one LLM run).
