# Supplementary material for: Natural Language Processing and Social Determinants of Health in Mental Health Research: AI-Assisted Scoping Review
Source: JMIR Ment Health. 2025 Jan 16;12:e67192. doi: 10.2196/67192 (PMC11756842; doi:10.2196/67192)
Supplement: Multimedia Appendix 2 [file mental-v12-e67192-s002.docx]

| *Phase of the review* | *LLM prompt* |
| --- | --- |
| Abstract screening | Summarize the text abstract of a full research paper (article), and given the below criteria list, say if the full paper is likely to be included, excluded, or unclear.  Criteria list.  Include: Paper should be using some kind of natural processing method (NLP), like transformers, pattern-matching, ChatGPT, GPT-3, BERT, Llama, Mistral, large language models, LDA/LSA, deep learning or machine learning applied to text, and similar.  Include: Paper should be in one of the mental health areas, such as: psychology, well-being, psychiatry, social work, substance abuse, marriage therapy, addiction therapy, suicide, grief, bereavement, trauma, stressful life events, counseling, or related. Cyberbullying and study of emotions should be included, however, aggressive and violent language should be excluded.  Exclude: If any of the Include criteria doesn't match  Exclude: Review papers (systematic, scoping, literature, narrative, and other type of reviews, but retrospective data reviews and chart reviews should be included), book chapters.  Exclude: Abstract is not provided, or it is too brief and doesn't contain enough information  Follow this format:  1) First provide some explanations why each study should be included or excluded.  2) Then format your output as follows, strictly follow this format, use equal(=) sign, if study is excluded, write 'answer=excluded', if study is included output 'answer=included', or if it is unclear write 'answer=unclear'. |
| Full-text screening | Look at the research paper (article), and given the below criteria list, say if the full paper is to be included, excluded, or unclear.  \nCriteria list.  Exclude reason 1: Doesn't use using some kind of natural language processing method (NLP), like transformers, pattern-matching, ChatGPT, GPT-3, BERT, Llama, Mistral, large language models (LLM), LDA/LSA, deep learning or machine learning applied to text, and similar.  Exclude reason 2: Not focused on one of the mental health areas, such as: psychology, well-being, psychiatry, social work, substance abuse, marriage therapy, addiction therapy, suicide, grief, bereavement, trauma, stressful life events, counseling,  Exclude reason 3: Review papers (systematic, scoping, literature, narrative, and other type of reviews, but retrospective data reviews and chart reviews should be included), conference papers, book chapters  Exclude reason 4: Not related to human health or well-being  Exclude reason 5: Full text is not provided, or it is too brief and doesn't contain enough information  \nFollow this format:  1) First provide some explanations why each study should be included or excluded.  2) Provide citation from text showing what NLP method was used and mental health problem explored.  3) Output the following, choose one best matching exclusion reason:  include=yes/no/unclear  exclude_reason=reason_number |
| Extraction of data | Definition of text dataset:  Text dataset (also called corpus, notes collection, notes database, text archive, text compilation, text repository, or similar term can be used) is a collection of texts or notes that were used for natural language processing (NLP) analysis or for training NLP models,  or for data extraction (also called text mining).  Look at the research paper (article), and extract the following information.  Follow the format given.\n  Field 1) Extract country and US state (if it is in US) where study location was. Typically it is the location where dataset comes from as described in the methods section. If this can not be determined, look at the country and US state of first author's affiliation. Output as: Country name, or USA/State Name  Field 2) What natural language processing (NLP) method was used (generally described in Methods section), example answers: the study didn't use natural language processing, word2vec, text2vec, doc2vec, RNN, CNN, SVM, random forest, deep learning, pattern-matching, ChatGPT, GPT-4, BERT, Llama, Mistral, LDA/LSA, other (provide name).  Field 3) What mental health problem(s) were investigated in the paper?  Field 4) What is the mental health area or specialty that best represents this paper, select one of: not related to mental health, psychology, well-being, psychiatry, social work, substance abuse, marriage therapy, addiction therapy, suicide, grief, bereavement, trauma, stressful life events, counseling, other (provide name).  Field 5) List all variables used in the study related to demographics, for example: age, race, ethnicity, gender, sex at birth, marital status, relationship status, sexual orientation, etc.  Field 6) List all variables used in the study related to social determinants of health, such as: none mentioned, urban/rural, transportation availability, access to healthcare, incarceration, income, poverty, health insurance, language knowledge, living arrangement, children/childless, family, adverse childhood experiences, housing, education, religion, stress, traumatic events, stressful life events, etc.  The next fields are all related to the text dataset that was used in the study:  Field 7) What is the name of the text dataset that was used for the Methods section (not to be confused with Introduction)  Field 8) What is the type of this text dataset, select one of: clinical notes, therapy session notes, social media platforms, online forum, other [insert type here]?  Field 9) What information or variables were extracted from this text dataset?  Field 10) Is it mentioned in the paper if it is possible for other researchers to get access to this text dataset?  Field 11) If it is mentioned in the paper that it is possible to get access to this text dataset, what kind of access it is? Select one of: public, public with restrictions, private, not given, not mentioned  Terms of access to text dataset can sometimes be found in the methods section, sometimes in data availability section, however this section has to specifically mention the text dataset that was used in this study. Sometimes terms of access are found in other parts of the document.  If the dataset can be found online or in well-known competition platforms like Kaggle consider access as public.  Field 12) If it is mentioned in the paper that access to this text dataset is public or public with restrictions, what is required to get access (can be training, signing use agreement, emailing the author, or similar)?  Field 13) Link (URL) to the text dataset, if provided.  Format your output as an R data.frame:  data.frame(fld1='',fld2='',fld3='',...,fld13='') |
