# Supplementary material for: Natural Language Processing and Social Determinants of Health in Mental Health Research: AI-Assisted Scoping Review
Source: JMIR Ment Health. 2025 Jan 16;12:e67192. doi: 10.2196/67192 (PMC11756842; doi:10.2196/67192)
Supplement: Multimedia Appendix 3 [file mental-v12-e67192-s003.docx]

*Table S1.* Benchmark of abstract screening phase (N=100 abstracts).

|  | Sensitivity | Specificity | Pos Pred Value | Neg Pred Value | Precision | Recall | F1 | Prevalence | Detection Rate | Detection Prevalence | Balanced Accuracy |
| --- | --- | --- | --- | --- | --- | --- | --- | --- | --- | --- | --- |
| Human reviewer vs Consensus | 0.89 | 0.91 | 0.92 | 0.87 | 0.92 | 0.89 | 0.91 | 0.54 | 0.48 | 0.52 | 0.9 |
| LLM vs Consensus | 0.98 | 0.96 | 0.96 | 0.98 | 0.96 | 0.98 | 0.97 | 0.54 | 0.53 | 0.55 | 0.97 |

*Table S2.* Benchmark of full-text screening phase (N=30 full-text PDFs).

|  | Sensitivity | Specificity | Pos Pred Value | Neg Pred Value | Precision | Recall | F1 | Prevalence | Detection Rate | Detection Prevalence | Balanced Accuracy |
| --- | --- | --- | --- | --- | --- | --- | --- | --- | --- | --- | --- |
| Human reviewer vs Consensus | 0.7 | 1 | 1 | 0.87 | 1 | 0.7 | 0.82 | 0.33 | 0.23 | 0.23 | 0.85 |
| LLM vs Consensus | 0.7 | 0.95 | 0.87 | 0.86 | 0.88 | 0.7 | 0.78 | 0.33 | 0.23 | 0.27 | 0.82 |

*Table S3.* Benchmark of full-text extraction phase (N=30 full-text PDFs).

|  | Country | NLP method | Mental health Outcome | Demographic variables | SDOH  Variables | Dataset name | Dataset type | Type of information extracted | Is access to dataset discussed? | Access level mentioned | Requirements to access dataset | URL to dataset | Average |
| --- | --- | --- | --- | --- | --- | --- | --- | --- | --- | --- | --- | --- | --- |
| Precision | 0.97 | 0.9 | 0.93 | 0.77 | 0.97 | 0.9 | 0.9 | 0.87 | 0.8 | 0.8 | 0.97 | 0.87 | *0.89* |
